# Therapeutic efficacy of telitacicept in a patient with GFAP autoimmune astrocytopathy: a case report

**DOI:** 10.3389/fimmu.2025.1691029

**Published:** 2025-10-16

**Authors:** Yan Lin, Zengyan Diao, Jianwei Low, Aibin Wang, Wei Wu, Lili Cao

**Affiliations:** ^1^ Department of Neurology, Qilu Hospital of Shandong University, Jinan, Shandong, China; ^2^ Department of Neurology, Liaocheng Second People’s Hospital, Liaocheng, Shandong, China

**Keywords:** GFAP-A, telitacicept, BLyS/APRIL, autoimmune meningoencephalomyelitis, B-cell targeted therapy

## Abstract

Glial fibrillary acidic protein autoimmune astrocytopathy (GFAP-A) represents a rare form of autoimmune meningoencephalomyelitis, characterized by the presence of GFAP-IgG antibodies. In most cases, GFAP-A shows a dramatic response to high-dose corticosteroids, and escalation to additional immunotherapy is rarely required. Relapses occur in approximately 20–30% of patients, particularly in those with severe phenotypes such as myelitis or with concomitant malignancy. Here, we present a patient with GFAP-A who initially presented with Epstein–Barr virus (EBV) detected in the cerebrospinal fluid (CSF) but negative GFAP-IgG and was considered to have viral encephalitis. As the condition progressively worsened, repeat testing revealed CSF GFAP-IgG positivity (1:32) and serum positivity (1:100), confirming the diagnosis of GFAP-A. High-dose glucocorticoids and intravenous immunoglobulin (IVIG) produced only limited benefit. The patient presented with fever, meningoencephalitic symptoms, limb weakness, orthostatic hypotension, and MRI abnormalities involving the brainstem and cervical spinal cord. Given the suboptimal response, telitacicept (240 mg, subcutaneously weekly for four weeks, followed by biweekly administration), a dual BLyS/APRIL inhibitor targeting B-cell maturation and plasma cell survival, was initiated. Treatment resulted in rapid symptom resolution, marked radiological and CSF improvement, and stable remission during follow-up. No severe adverse events were observed. This case highlights telitacicept as a promising and well-tolerated therapeutic option for refractory GFAP-A.

## 1 Introduction

Glial fibrillary acidic protein autoimmune astrocytopathy (GFAP-A) is a recently recognized autoimmune meningoencephalomyelitis, first reported by Fang et al. in 2016 (1, 2). It is characterized by the presence of GFAP-immunoglobulin G (GFAP-IgG), most specifically detected in cerebrospinal fluid (CSF), and presents clinically with acute or subacute meningoencephalitis symptoms such as fever, headache, altered mental status, seizures, and occasionally myelitis or optic neuritis (3, 4). Magnetic resonance imaging (MRI) can display a radial perivascular enhancement pattern in the brain, although this finding is not universal; when present together with CSF GFAP-IgG positivity, it strongly supports the diagnosis (5, 6).

Although high-dose glucocorticoids and intravenous immunoglobulin (IVIG) are considered first-line therapies, some patients subsequently receive immunosuppressive agents such as mycophenolate mofetil, azathioprine, or rituximab, with variable efficacy. While these agents can be effective in certain cases, others show suboptimal responses (2, 7, 8). Pathological studies have demonstrated perivascular inflammatory infiltrates predominantly composed of lymphocytes and plasma cells with astrocytic injury, suggesting a central role of B-cell activation and autoantibody production in disease pathogenesis (9). Case reports have further shown that ofatumumab, a fully human anti-CD20 monoclonal antibody, may induce remission in patient refractory to rituximab, underscoring the potential of B-cell–targeted therapy (10). A multicenter cohort study from China reported that nearly 40% of GFAP-A patients developed relapses within two years, emphasizing the unmet need for more effective long-term immunomodulatory strategies (11). In this context, telitacicept, a dual BLyS/APRIL inhibitor that modulates both B-cell maturation and plasma cell survival, may represent a novel therapeutic option for relapsing or refractory GFAP-A.

Telitacicept is an innovative recombinant fusion protein, consisting of the extracellular domain of the transmembrane activator and CAML interactor (TACI) linked to the Fc fragment of human IgG. In March 2021, it received approval in China for the treatment of active systemic lupus erythematosus (12). As a dual inhibitor of B lymphocyte stimulator (BLyS) and a proliferation-inducing ligand (APRIL)—two critical cytokines involved in B-cell maturation and plasma cell survival—telitacicept effectively reduces autoantibody production. Beyond its application in lupus, telitacicept has demonstrated therapeutic potential across a wide range of antibody-mediated autoimmune diseases, including IgA nephropathy, multiple sclerosis, myasthenia gravis (MG), neuromyelitis optica spectrum disorder (NMOSD), rheumatoid arthritis, Sjögren’s syndrome, and granulomatosis with polyangiitis (13–17). These findings underscore its promise as a well-tolerated, B-cell–targeted therapeutic agent. Nonetheless, its potential efficacy in autoimmune neurological disorders, such as GFAP-A, remains unexplored.

Here, we describe a patient with progressive GFAP-A who demonstrated marked clinical improvement and sustained disease stabilization following treatment with telitacicept after failure of conventional therapies. This case provides preliminary evidence supporting dual BLyS/APRIL blockade as a potential therapeutic strategy for refractory GFAP-A and underscores the importance of exploring B-cell–targeted approaches in this rare but challenging autoimmune astrocytopathy.

## 2 Case presentation

A 39-year-old man was admitted to our hospital with a 2-week history of recurrent fever, headache and limb weakness. Neurological examination revealed somnolence, neck stiffness, positive Kernig’s sign, and bilateral lower limb weakness graded as 4−/5. No visual impairment or seizures were noted. He had no significant past medical history or family history of autoimmune disease. On admission, he presented with concomitant pulmonary infection, and he also had a history of allergic predisposition.

At the first lumbar puncture, cerebrospinal fluid (CSF) analysis showed lymphocytic pleocytosis (100 cells/mm³, 92% lymphocytes), elevated protein (0.83 g/L), and normal glucose, with an elevated lactate of 4.7 mmol/L. Oligoclonal bands (OCBs) were type I, and metagenomic next-generation sequencing (mNGS) detected Epstein–Barr virus (EBV). At that time, GFAP-IgG was negative, and the clinical picture was initially considered consistent with viral encephalitis. The patient therefore received antiviral and anti-infective therapy.

**Figure 1:**
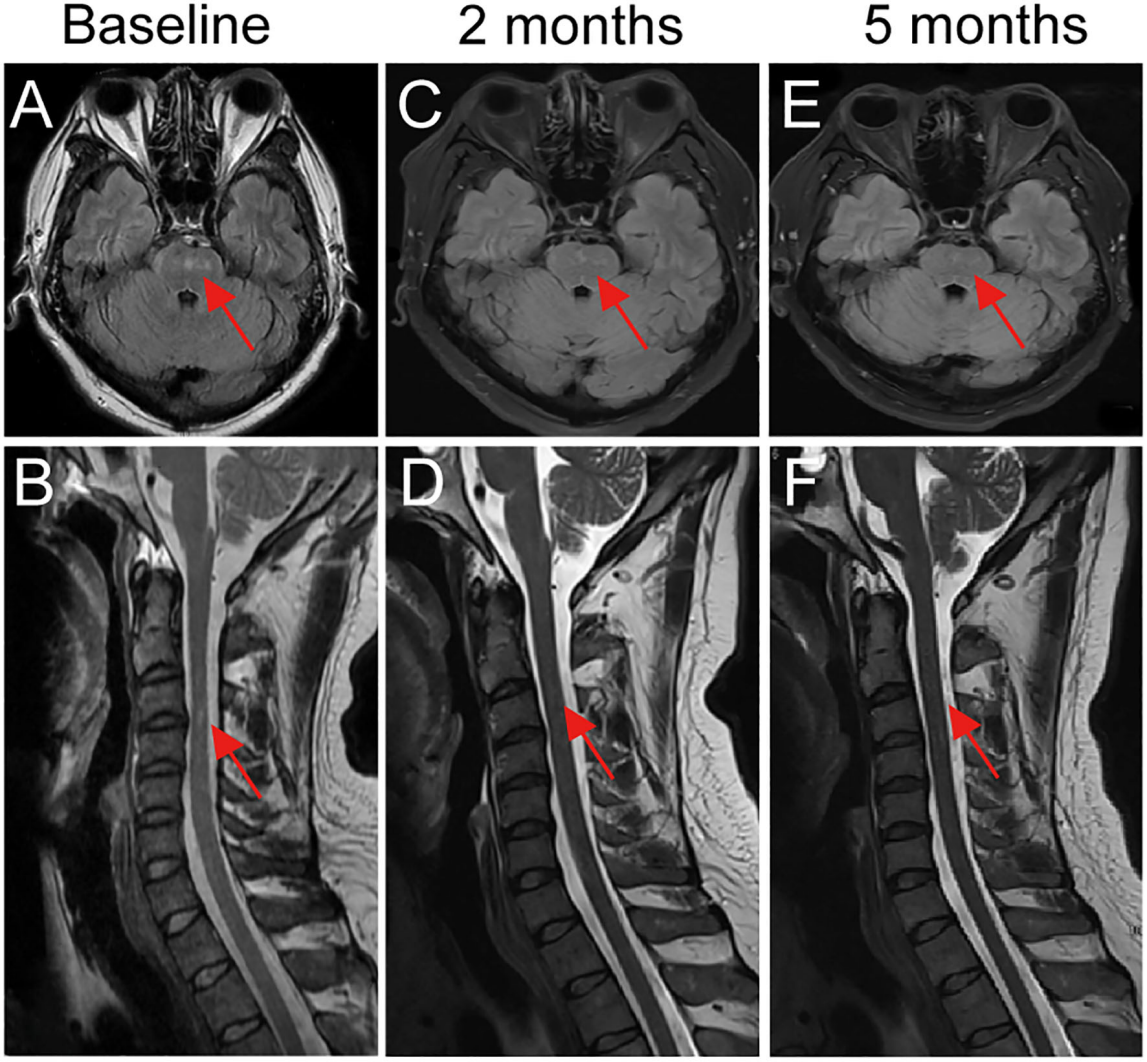
Longitudinal brain and spinal MRI changes in a patient with GFAP- A treated with telitacicept. **(A, B)** Baseline MRI revealed hyperintense lesions in the pons and cervicothoracic spinal cord. **(C, D)** After 2 months of telitacicept therapy, lesions showed marked reduction in size and signal intensity in both the brainstem and spinal cord. **(E, F)** At 5 months, complete resolution of brainstem and spinal cord lesions was observed, consistent with sustained radiological remission. Red arrows indicate lesion sites.

However, as the patient’s symptoms progressively worsened, a repeat lumbar puncture was performed, which revealed GFAP-IgG positivity in both CSF (1:32) and serum (1:100), while OCBs converted to type II. Brain MRI demonstrated abnormal signals in the pons, bilateral middle cerebellar peduncles, and cervicothoracic spinal cord (**Figures 1A, B**). Serum and CSF were negative for aquaporin-4 (AQP4), myelin oligodendrocyte glycoprotein (MOG), and myelin basic protein (MBP) antibodies, as well as serum ganglioside and onconeural antibodies. In addition, autoimmune panels were performed to exclude connective tissue diseases and central nervous system vasculitis, all of which were negative. To rule out paraneoplastic causes, the patient underwent chest, abdominal, and pelvic CT scans as well as serum tumor marker testing, and no abnormalities were detected. Based on these findings, a diagnosis of GFAP autoimmune astrocytopathy (GFAP-A) was established.

The patient initially received intravenous methylprednisolone 500 mg/day for 5 days, followed by 240 mg/day for 3 days and 120 mg/day for 3 days, then transitioned to oral prednisone 60 mg/day (1 mg/kg/day). Intravenous immunoglobulin (IVIG, 0.4 g/kg/day for 5 days) was administered in parallel. However, despite corticosteroid pulse therapy and IVIG, his symptoms continued to progress, including persistent meningoencephalitic features and autonomic dysfunction. Given this progression and limited response to conventional therapy, telitacicept was initiated at 240 mg subcutaneously once weekly, adjusted to every 2 weeks after 4 weeks.

**Figure 2:**
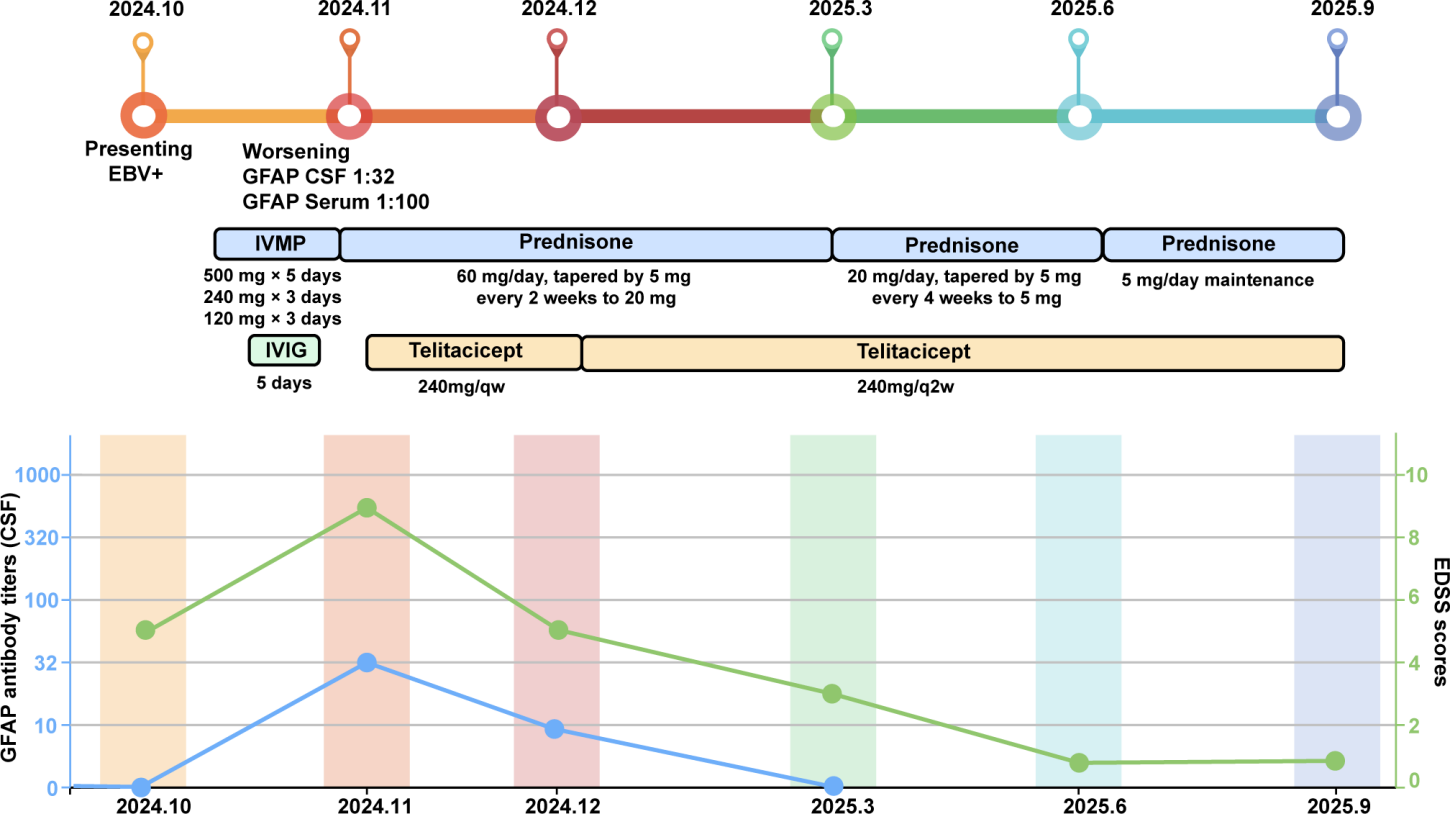
Schematic illustration of the disease course showing therapeutic interventions, changes in Expanded Disability Status Scale (EDSS) scores, and GFAP-IgG antibody titers.

Following telitacicept treatment, symptoms rapidly improved, with resolution of fever and headache, relief of orthostatic hypotension, and no further limb weakness or numbness. Oral prednisone was tapered by 5 mg every 2 weeks until 20 mg/day, then by 5 mg monthly, and maintained at 5 mg/day thereafter. At the 2-month follow-up, CSF analysis revealed GFAP-IgG positivity at lower titers (1:10), while serum GFAP-IgG became negative, and repeat brain MRI demonstrated significant reduction of lesions (**Figures 1C, D**). At 5-months of follow-up, brain MRI showed complete resolution of lesions (**Figures 1E, F**), and CSF GFAP-IgG was negative. At 8 months of follow-up under telitacicept, the patient remained clinically stable with no further disease progression, and no severe adverse events were observed. To better illustrate the temporal relationship between therapeutic interventions and clinical changes, a flow chart is provided (**Figure 2**).

## 3 Discussion

GFAP-A is generally characterized by a dramatic response to corticosteroid therapy, and relapses occur in approximately 20–30% of patients, particularly in those with severe phenotypes such as myelitis or with concomitant malignancy (1, 18). In our case, the patient represented an exception, showing progressive disease despite corticosteroid pulse therapy and IVIG. He presented with typical clinical features including fever, headache, altered consciousness, and brainstem and spinal cord involvement, together with autonomic dysfunction manifested as orthostatic hypotension. Importantly, metagenomic sequencing initially detected EBV in the cerebrospinal fluid when GFAP-IgG was still negative, and GFAP-IgG positivity emerged only as the disease worsened. This temporal relationship suggests that viral infection may have acted as a trigger or aggravating factor for GFAP autoimmunity, consistent with previous reports linking the disease to parainfectious or paraneoplastic phenomena (19–21).

Therapeutically, the patient experienced only temporary improvement with high-dose intravenous methylprednisolone and IVIG, but subsequently relapsed, exhibiting additional autonomic dysfunction. This pattern underscores the refractory and relapsing nature of the disease. Conventional immunosuppressants have demonstrated limited efficacy in comparable cases, and the application of rituximab is frequently hindered by incomplete B-cell depletion or infusion-related allergic reactions (22, 23). In this instance, following the ineffectiveness of standard therapies, telitacicept was administered, resulting in rapid clinical recovery within four weeks. MRI lesions showed progressive regression leading to complete resolution, and CSF GFAP-IgG titers declined to negativity. Over an eight-month follow-up period, the patient remained stable without experiencing a relapse. This clinical course underscores the broader spectrum of B-cell and plasma cell suppression achieved through dual BLyS/APRIL blockade, which may address the limitations associated with CD20-targeted monotherapy.

Telitacicept has shown promising efficacy across a range of B-cell–mediated autoimmune diseases (12). It is approved in China for the treatment of SLE, where randomized trials have demonstrated reductions in autoantibody titers and clinical activity (15). Preliminary studies have further explored its potential in neuroimmunological conditions: in NMOSD, telitacicept can prolong relapse-free intervals, lower annualized relapse rates, and improve disability scores, with concurrent reductions in MRI lesion burden and AQP4 antibody titers (17, 24). Similarly, in MG, randomized controlled data demonstrated rapid and sustained improvements in QMG scores, accompanied by consistent reductions in serum immunoglobulin levels, with a favorable safety and tolerability profile (16, 25). Beyond neuroimmunology, case reports have described clinical benefit in refractory granulomatosis with polyangiitis and autoimmune-related hepatitis, further supporting its broad immunomodulatory potential across systemic and organ-specific autoimmune diseases (26, 27).

Despite the encouraging outcome in our patient, this report is limited by being a single-case study. The optimal dosing regimen (weekly vs. biweekly), treatment duration, and long-term safety remain undefined. Additionally, the immunopathological mechanisms underlying telitacicept’s effect in GFAP-A—particularly its impact on B-cell subsets, autoantibody kinetics, and CNS immune microenvironment—require further clarification. Future directions should include multicenter prospective studies or randomized controlled trials to validate clinical efficacy, determine relapse-prevention capacity, and refine therapeutic parameters. Parallel mechanistic investigations combining immunophenotyping and biomarker profiling may also help identify predictors of response and guide individualized treatment strategies.

## Data Availability

The original contributions presented in the study are included in the article/supplementary material. Further inquiries can be directed to the corresponding authors.

## References

[1] Boyan F Andrew M Shannon RH Thomas JK Sean JP Allen JA . Autoimmune glial fibrillary acidic protein astrocytopathy: A novel meningoencephalomyelitis. JAMA Neurol. (2016) 73:1297–307. doi: 10.1001/jamaneurol.2016.2549, PMID: 27618707

[2] Amy K Anastasia Z Andrew M . Autoimmune glial fibrillary acidic protein astrocytopathy. Curr Opin Neurol. (2019) 32:452–8. doi: 10.1097/wco.0000000000000676, PMID: 30724768 PMC6522205

[3] Eoin PF Shannon RH Vanda AL Boyan F Allen JA P Pearse M . Glial fibrillary acidic protein immunoglobulin G as biomarker of autoimmune astrocytopathy: Analysis of 102 patients. Ann Neurol. (2017) 81:298–309. doi: 10.1002/ana.24881, PMID: 28120349

[4] Fulan S Youming L Wei Q . Autoimmune glial fibrillary acidic protein astrocytopathy: A review of the literature. Front Immunol. (2018) 9. doi: 10.3389/fimmu.2018.02802, PMID: 30568655 PMC6290896

[5] Runhua B Li A Wei D Zhiwei W Xiaokun Q Jianguo L . Autoimmune glial fibrillary acidic protein astrocytopathy misdiagnosed as intracranial infectious diseases: case reports and literature review. Front Immunol. (2025) 16. doi: 10.3389/fimmu.2025.1519700, PMID: 39911384 PMC11794125

[6] Caroline H Russell O Eoin PF Dagur IJ Fredrik P Brenda B . Clinical and neuroimaging phenotypes of autoimmune glial fibrillary acidic protein astrocytopathy: A systematic review and meta-analysis. Eur J Neurol. (2024) 31:e16284. doi: 10.1111/ene.16284, PMID: 38506182 PMC11235751

[7] Yuto M Takanori H Sho N Kazumasa S Mai T Tsubasa W . Case report: Atypical case of autoimmune glial fibrillary acidic protein astrocytopathy following COVID-19 vaccination refractory to immunosuppressive treatments. Front Immunol. (2024) 15. doi: 10.3389/fimmu.2024.1361685, PMID: 38665914 PMC11043467

[8] Ahmed S VCC K Kannoth S Raeesa F Misri Z Mascarenhas DG . Autoimmune GFAP astrocytopathy-beyond the known horizon, India's first multifaceted institutional experience. Ann Neurosci. (2025) 32:248–58. doi: 10.1177/09727531241230213, PMID: 39544637 PMC11559843

[9] Guo Y Endmayr V Zekeridou A McKeon A Leypoldt F Hess K . New insights into neuropathology and pathogenesis of autoimmune glial fibrillary acidic protein meningoencephalomyelitis. Acta Neuropathol. (2024) 147:31. doi: 10.1007/s00401-023-02678-7, PMID: 38310187 PMC10838242

[10] Cao S Du J Pan S Zhang J Xu S Wei L . Case Report: Ofatumumab treatment in a patient with rituximab-intolerant refractory autoimmune GFAP astrocytopathy. Front Immunol. (2023) 14:1164181. doi: 10.3389/fimmu.2023.1164181, PMID: 37223100 PMC10200986

[11] Ti W Hao Z Chao G Qiuhua Y Moli F Lin-Jie Z . Glial fibrillary acidic protein astrocytopathy based on a two-center chinese cohort study. Ann Clin Transl Neurol. (2025) 12 (9):1813–22. doi: 10.1002/acn3.70118, PMID: 40641094 PMC12455873

[12] Dhillon S . Telitacicept: first approval. Drugs. (2021) 81:1671–5. doi: 10.1007/s40265-021-01591-1, PMID: 34463932

[13] Cai J Gao D Liu D Liu Z . Telitacicept for autoimmune nephropathy. Front Immunol. (2023) 14:1169084. doi: 10.3389/fimmu.2023.1169084, PMID: 37342346 PMC10277628

[14] Zeng L Yang K Wu Y Yu G Yan Y Hao M . Telitacicept: A novel horizon in targeting autoimmunity and rheumatic diseases. J Autoimmun. (2024) 148:103291. doi: 10.1016/j.jaut.2024.103291, PMID: 39146891

[15] Wu D Li J Xu D Merrill JT van Vollenhoven RF Liu Y . Telitacicept in patients with active systemic lupus erythematosus: results of a phase 2b, randomised, double-blind, placebo-controlled trial. Ann Rheum Dis. (2024) 83:475–87. doi: 10.1136/ard-2023-224854, PMID: 38129117 PMC10958275

[16] Yin J Zhao M Xu X Zhang M Xu Z Li Z . A multicenter, randomized, open-label, phase 2 clinical study of telitacicept in adult patients with generalized myasthenia gravis. Eur J Neurol. (2024) 31:e16322. doi: 10.1111/ene.16322, PMID: 38726639 PMC11235933

[17] Ding J Jiang X Cai Y Pan S Deng Y Gao M . Telitacicept following plasma exchange in the treatment of subjects with recurrent neuromyelitis optica spectrum disorders: A single-center, single-arm, open-label study. CNS Neurosci Ther. (2022) 28:1613–23. doi: 10.1111/cns.13904, PMID: 35851754 PMC9437241

[18] Gklinos P Athanasopoulos F Giatrakou V Arkoudis NA Pournara D Giagkou E . Unveiling GFAP astrocytopathy: insights from case studies and a comprehensive review of the literature. Antibodies (Basel). (2024) 13:79. doi: 10.3390/antib13040079, PMID: 39449321 PMC11503365

[19] Yu X Zou Y Li M Wang L Feng W Wei L . Auto-immune glial fibrillary acidic protein astrocytopathy with active intrathecal epstein-barr virus: A single-center case series report. Neuropsychiatr Dis Treat. (2025) 21:1119–30. doi: 10.2147/NDT.S483073, PMID: 40469537 PMC12136066

[20] Wang C Zhang H Lu W Zhan Y . The EBV connection: a severe case of GFAP-A with central hypoventilation unresponsive to IVIG and literature review. Eur J Med Res. (2024) 29:415. doi: 10.1186/s40001-024-01926-0, PMID: 39135139 PMC11320868

[21] Li XL Wang JY Li LK Yang CL Zhao XL Yang B . Epstein-Barr virus: To be a trigger of autoimmune glial fibrillary acidic protein astrocytopathy? CNS Neurosci Ther. (2023) 29:4139–46. doi: 10.1111/cns.14336, PMID: 37458208 PMC10651959

[22] Fouda GE Bavbek S . Rituximab hypersensitivity: from clinical presentation to management. Front Pharmacol. (2020) 11:572863. doi: 10.3389/fphar.2020.572863, PMID: 33013416 PMC7508176

[23] Polito V Barbacki A Isabwe G . Type I allergic reaction to rituximab upon first lifetime exposure: a case report. Allergy Asthma Clin Immunol. (2020) 16:56. doi: 10.1186/s13223-020-00448-8, PMID: 32607108 PMC7318363

[24] Dai M Huang C Zhou M Leong PY Chen X . Dual BLyS/APRIL targeted therapy with telitacicept in rituximab-refractory SLE-associated neuromyelitis optica spectrum disorder: a case report. Front Immunol. (2025) 16:1602800. doi: 10.3389/fimmu.2025.1602800, PMID: 40491913 PMC12146289

[25] Lin J Li Y Gui M Bu B Li Z . Effectiveness and safety of telitacicept for refractory generalized myasthenia gravis: a retrospective study. Ther Adv Neurol Disord. (2024) 17:17562864241251476. doi: 10.1177/17562864241251476, PMID: 38751755 PMC11095194

[26] Huang L Lin W Liu Y Zhu J Li Y Zheng Z . Combination treatment with telitacicept, cyclophosphamide and glucocorticoids for severe Granulomatous polyangiitis: a case report and literature review. Front Immunol. (2023) 14:1298650. doi: 10.3389/fimmu.2023.1298650, PMID: 38106422 PMC10722187

[27] Wang W Ma X Zhang B Zhang Z Wu X Jiang H . Case Report: A refractory unusual tetrad of overlap syndrome involving rheumatoid arthritis, Sjögren's syndrome, autoimmune hepatitis, and type 1 renal tubular acidosis, successfully treated with a BLyS/APRIL dual inhibitor. Front Immunol. (2025) 16:1558059. doi: 10.3389/fimmu.2025.1558059, PMID: 40170868 PMC11959057

